# Can a single model explain both breast cancer and prostate cancer?

**DOI:** 10.1186/1742-4682-4-28

**Published:** 2007-08-01

**Authors:** A Edward Friedman

**Affiliations:** 1 Department of Mathematics, University of Chicago, 5734 S. University Avenue, Chicago, IL 60637, USA

## Abstract

**Background:**

The Estradiol-Dihydrotestosterone model of prostate cancer (PC) showed how the interaction of hormones with specific hormone receptors affected apoptosis. The same hormone can produce different effects, depending on which hormone receptor it interacts with.

**Model:**

This model proposes that the first step in the development of most PC and breast cancer (BC) occurs when aromatase converts testosterone to estradiol (E2). A sufficiently high enough local level of E2 results in telomerase activity. The telomerase activity allows cell division and may lead to BC or PC, which will proliferate if the rate of cell division is greater than the rate of cell death. The effect of hormones on their hormone receptors will affect the rate of cell death and determine whether or not the cancer proliferates.

**Conclusion:**

By minimizing bcl-2 and maximizing apoptotic proteins, new systemic treatments for BC and PC can be developed that may be more effective than existing treatments.

## Background

The Estradiol-Dihydrotestosterone (E-D) model [1] of prostate cancer (PC) describes how PC works at the level of hormone receptors. In this model, no hormone is "good" or "bad", but the effect of each hormone is determined by its interaction with its hormone receptors. Each hormone receptor has an effect on apoptosis, or programmed cell death. Table 1 summarizes this model, with ↑ representing upregulation and ↓ representing downregulation. Although the exact mechanism of how the intracellular androgen receptor (iAR) is able to counter the effects of the membrane androgen receptor (mAR) is not known, for diagrammatic purposes, the process is represented in Table 1 as downregulation. This model can be expanded and extended to encompass breast cancer (BC) as well.

**Table 1:** E-D model of prostate cancer

**Hormone receptor**	**Property**
Membrane androgen receptor	↑apoptotic proteins ↑bcl-2 ↑calreticulin
Intracellular androgen receptor	↓apoptotic proteins ↓bcl-2
Estrogen receptor-αβ heterodimer	↑telomerase activity
Estrogen receptor-α	↑bcl-2
Estrogen receptor-β	↓bcl-2

## Model

### Model description

Aromatase (Aro) is an enzyme which converts testosterone (T) to estradiol (E2). If the Aro activity is high enough, a process is started that may result in BC or PC. High local levels of E2 result in human telomerase production and activity. If the rate of growth (R_G_) is greater than the rate of cell death (R_D_), then these cells will proliferate and cancer may result. Telomerase activity was sufficient to transform human cell lines that ordinarily have limited life spans into immortalized cell lines [2].

This model makes the assumption that the effects of hormones on hormone receptors are the same for BC and PC unless there is evidence to the contrary. Table 2 shows the properties of the hormone receptors as proposed in the extended E-D model.

**Table 2:** Extended E-D model of breast cancer and prostate cancer

**Hormone receptor**	**Property**
Membrane androgen receptor	↑apoptotic proteins ↓bcl-2 (BC only) ↑bcl-2 (PC only) ↓AS3 ↑Ca^++ ^influx
Intracellular androgen receptor	↓apoptotic proteins ↓bcl-2 ↑AS3 ↓Ca^++ ^influx ↑calreticulin
Estrogen receptor-αβ heterodimer	↑telomerase activity (PC only)
Estrogen receptor-α homodimer	↑telomerase activity
Estrogen receptor-α	↑bcl-2
Estrogen receptor-β	↓bcl-2
Membrane estrogen receptor	↑bcl-2
Progesterone receptor A	↑bcl-2
Progesterone receptor B	↓bcl-2
Membrane progesterone receptor	↓bcl-2

### Estrogen receptors

E2 upregulated both human telomerase mRNA and human telomerase activity in normal prostate epithelial cells, benign prostate hyperplasia, and the PC cell lines LNCaP, DU145, and PC-3 [3]. In the presence of E2, a vector that resulted in the overproduction of estrogen receptor-α (ER-α) showed an increase in telomerase promoter activity for PC and for the BC cell line MCF-7. However, in the presence of E2, a vector that resulted in the overproduction of ER-β showed an increase in telomerase promoter activity in PC, but not in BC. Increasing ER-α would result in an increase in ER-α homodimers, a decrease in ER-β homodimers, and an increase in ER-αβ heterodimers. Similarly, increasing ER-β would result in an increase in ER-β homodimers, a decrease in ER-α homodimers, and an increase in ER-αβ heterodimers. This is all consistent with ER-αβ heterodimers upregulating telomerase activity in prostate epithelial cells and PC. However, another possibility is that both ER-α homodimers and ER-β homodimers upregulate telomerase activity. If heterodimers were not involved, then ER-α should not be needed to increase telomerase activity. However, mice lacking ER-α do not develop PC [4]. Assuming that the reason for this is that without ER-α, no telomerase activity could occur in the prostate epithelial cells, then this would be consistent with ER-αβ heterodimers upregulating telomerase activity. It is still possible that ER-α homodimers could upregulate telomerase activity as well. When 4-hydroxytamoxifen (OHT) was added to LNCaP cells transfected with the expression vector for ER-α, telomerase activity was upregulated, but not when transfected with the expression vector for ER-β instead [3]. This is consistent with OHT upregulating telomerase activity in PC by acting as an agonist for ER-α homodimers. The extended E-D model takes the view that ER-α homodimers are responsible for the increase in telomerase activity in BC and PC because if ER-α receptors alone were able to increase telomerase activity, then ordinary levels of E2 might lead to telomerase activity. The important point is that for both BC and PC, a local increase in the level of E2 results in an increase in telomerase activity.

One of the requirements for any cancer to grow is limitless replicative potential [5]. Ordinarily, cells are capable of a limited number of divisions due to their telomere length, which shortens following each division. Cell division in the absence of sufficient telomere length usually results in senescence or apoptosis due to accumulation of the apoptotic protein p53 [6]. Mutations in p53 allow cell division to occur in the absence of sufficient telomere length, but usually result in chromosomal instability that may lead to carcinogenesis [7]. Telomeres can be lengthened by telomerase activity or by alternative lengthening of telomeres (ALT) [8]. Telomerase activity has been found in 90% of prostate carcinomas and 88% of ductal and lobular breast carcinomas [9]. This is consistent with telomerase activity being one of the first steps in almost all BC and PC. Those without telomerase activity would be expected to have ALT or mutations in p53.

In disease-free breast adipose tissue, Aro activity is usually expressed at low levels due to promoter I.4 [10]. In adipose tissue of BC, Aro activity is much higher due to the presence of promoters I.3 and II. Cyclic adenosine 3',5'-monophosphate (cAMP) analogues switch the promoters to I.3 and II for human adipose fibroblasts (HAFs) [11]. Exposing HAFs to BC cell-conditioned medium induced promoter II activity in a process independent of cAMP [10]. Cell-conditioned media of normal breast epithelial cells, liver cancer cells, and PC cells all failed to induce promoter II activity in HAFs. This is consistent with one or more factors found in BC being responsible for the increased Aro activity in HAFs. E2 was found in significantly higher concentrations in BC than in normal breast tissue [12]. The level of E2 found in the BC of postmenopausal women was similar to that found in the BC of premenopausal women. The local level of E2 was 10 times higher for BC in postmenopausal women than the level found in their blood plasma or normal breast tissue [13]. This is consistent with most BC starting due to the production of one or more factors in the breast epithelial cells which have the capability of inducing promoter II activity in the surrounding adipose tissue. More research is needed to discover how promoter I.3 activity is induced and to learn what factors are responsible for inducing promoter II activity.

Aro activity was not observed [14] in normal prostate epithelial cells, but was observed in the PC cell lines LNCaP, DU145, and PC-3. The level of Aro activity in PC was in the same range as Aro activity in BC. Mice lacking the Aro gene never develop PC. Also, Aro activity was detected [15] in three of four PC tumors that were tested. The occasional PC tumor lacking Aro activity can be explained by the PC having ALT, mutated p53, or a mutation that promotes telomerase activity without requiring Aro activity. These findings are consistent with most PC starting due to the permanent activation of the Aro gene.

ER-α and ER-β are known to tend to counteract each other [16]. E2 increased the production of bcl-2 in MCF-7 [17], an ER-α positive cell line of BC. This increase was negated by the addition of OHT, a known antagonist to ER-α in breast tissue [18]. This is consistent with ER-α being responsible for upregulating bcl-2. By applying the principle of ER-β acting in opposition to ER-α, then ER-β should downregulate bcl-2 in BC.

Mice with a genetic mutation that knocks out ER-β have an overexpression of bcl-2 in their ventral prostate [19]. This is consistent with ER-β downregulating bcl-2 in PC. In accordance with the principle of ER-α acting in opposition to ER-β, then ER-α should upregulate bcl-2 in PC.

Membrane estrogen receptor (mER) upregulated bcl-2 in the BC line T47D [20]. All of the above is consistent with mER and ER-α upregulating bcl-2 and ER-β downregulating bcl-2. More research is needed on the specific hormone receptors to verify and to quantify these findings.

### Progesterone receptors

Mifepristone (RU-486), a drug that is antagonistic to progesterone receptor A (PRA), decreased bcl-2 production in LNCaP [21], an androgen dependent PC (ADPC) cell line. Production of bcl-2 was decreased even further when progesterone (P) was added in addition to RU-486. This is consistent with PRA upregulating bcl-2 and either progesterone receptor B (PRB), membrane progesterone receptor (mPR), or both, downregulating bcl-2. However, further experiments must be done on other cell lines, since LNCaP has been shown to have mutated iAR that binds to P [22] and iAR downregulates bcl-2. The extended E-D model takes the view that both PRB and mPR downregulate bcl-2 in PC, but further experimentation must be done to verify this.

The mutations BRCA1 and BRCA2 have a striking lack of PRB expression in normal breast cells [23]. BRCA1 mutations result in an increased chance of developing BC for women, but not men, and an increased chance of developing PC for men. BRCA2 mutations result in an increased chance of developing BC for men and for women and an increased chance of developing PC for men [24]. According to the extended E-D model, the decreased amount of PRB should result in decreased downregulation of bcl-2, resulting in higher levels of bcl-2 being present. While this would not in itself cause BC or PC, it does increase the likelihood that R_G _> R_D _if an initial cancer cell arises.

The fact that men with BRCA1 mutations do not have an increased chance of developing BC can be explained by the decreased downregulation of bcl-2 that results from the loss of PRB being offset by men's high levels of T which results in both mAR and iAR significantly downregulating bcl-2. If a high enough level of T is present, it is possible that no net increase in bcl-2 would occur in spite of the absence of PRB. In PC, high levels of T end up with iAR downregulating bcl-2, but with mAR upregulating bcl-2, which results in more bcl-2 being present than is the case for BC. Since women have a much lower level of T than men do, the increase in bcl-2 that results from the loss of PRB would probably not be offset by the downregulation of bcl-2 by the androgen receptors. This is consistent with BRCA1 increasing the level of bcl-2 in the breast tissue of women but not of men. Since BRCA2 mutations increase the chance of developing BC for both men and women, this implies that there is another factor present in BRCA2 mutations which decreases R_D _or increases R_G _in addition to the elimination of PRB. Further research is needed to clarify this point.

Mice which were BRCA1/p53 deficient all developed BC, unless they were treated with RU-486, in which case none of the mice developed BC [25]. This is consistent with bcl-2 production increasing in response to P for BRCA1 mutations due to PRA upregulating bcl-2 and the absence of PRB downregulating bcl-2. Therefore, the fact that BC proliferated in the absence of PRB means that R_G _> R_D_. The fact that RU-486 prevented BC development is consistent with mPR downregulating bcl-2. This is because in the presence of RU-486, there is no PRA or PRB available for P to bind to, and since this prevents BC, then R_G _< R_D_. If mPR upregulated bcl-2, then P would have caused an increase in bcl-2, which might have resulted in some of the mice developing BC if R_D _became low enough. Assuming mPR downregulates bcl-2, but not as strongly as PRA upregulates bcl-2, then in the absence of RU-486, P would have resulted in an increase in bcl-2 and therefore an increased incidence of BC due to the decrease in R_D_, whereas in the presence of RU-486, P would have resulted in a decrease in bcl-2 and therefore a decreased incidence of BC due to the increase in R_D_, which what was in fact observed. Also, it is likely that the combined BRCA1/p53 deficiency still resulted in the same number of initial BC cells arising in all of the mice, but since R_G _< R_D _in the presence of RU-486, the BC was unable to proliferate.

The inferences drawn by combining the above experiments are consistent with the conclusion of the extended E-D model that PRA upregulates bcl-2, whereas PRB and mPR downregulate bcl-2. However, further testing is needed to conclusively prove these points.

### Androgen receptors

By using T-BSA, which is known to bind to mAR but not to iAR, it was shown that mAR upregulates [26] bcl-2 in PC, but downregulates [20] it in BC. mAR upregulated the apoptotic protein Bad in BC [20] and Fas in PC [26]. Also, upregulation of the apoptotic proteins U19 and ALP1 in PC has been attributed to mAR due to the rapidity of their production immediately after androgen deprivation therapy (ADT) is ended [1]. For both BC and PC, flutamide, an antagonist of iAR, was used as a control. The effect of T-BSA on mAR was the same in the presence and in the absence of flutamide, further confirming that T-BSA bound to mAR but not to iAR. In both BC and PC, mAR exhibited rapid steroid effects typical of non-genomic steroid hormone actions, whereas iAR exhibited the slow effects typical of genomic steroid hormone actions, which typically take hours.

5α-dihydrotestosterone (DHT) downregulated bcl-2 in the PC cell line LNCaP-FGC [27] and in the BC cell line ZR-75-1 [28]. This downregulation disappeared when an antagonist of iAR was added. All of this is consistent with iAR downregulating bcl-2.

Androgens inhibited cell proliferation in the BC cell line MCF7-AR1 [29], which has approximately five times more iAR than the BC cell line MCF7 does. In the presence of androgens plus bicalutamide, an antagonist to iAR, no inhibition of cell proliferation was observed. Androgens upregulated AS3, a protein which shuts off cell proliferation, in MCF7-AR1 [30]. This is consistent with iAR upregulating AS3.

The PC cell line LNCaP-FGC has high levels of iAR [31]. High physiological levels of androgens caused proliferative shutoff of LNCaP-FGC [30]. There was a strong correlation between this proliferative shutoff and AS3 expression. The PC cell line LNCaP 104-R2 had its growth inhibited by T, but stimulated by T plus finasteride (F) [32]. F inhibits 5-α reductase type II (5AR2), which is an enzyme that converts T to DHT. LNCaP 104-R2 also has high levels of iAR [33]. This is all consistent with iAR upregulating AS3 in BC and PC. If mAR downregulates AS3, and ordinarily there is a balance between iAR and mAR, then it would require an imbalance that results in an overexpression of iAR with regards to mAR in order for AS3 to be upregulated. This would explain why LNCaP 104-R2 upregulated AS3 after being exposed to T. The high levels of iAR would have created the imbalance that led to AS3 being upregulated. When F was added, DHT conversion from T was blocked, so instead of DHT, T became the ligand for iAR. Since T binds to iAR with an affinity five times less than that of DHT [34], the necessary imbalance was no longer present and inhibition of growth no longer occurred. Further research is needed to determine exactly what effect mAR has on AS3 production.

Calcium ion (Ca^++^) influx increased when T-BSA was added to PC cells [35]. The observed increase in Ca^++ ^influx is consistent with mAR upregulating Ca^++ ^influx, since T-BSA binds to mAR but not to iAR. The fact that T-BSA caused Ca^++ ^influx, whereas T does not, is consistent with iAR downregulating Ca^++ ^influx. Ca^++ ^influx also occurs during ADT [36]. If the absence of androgen allows Ca^++ ^influx to occur, then it is likely that one or more proteins are responsible for preventing Ca^++ ^influx. This is consistent with iAR upregulating proteins which are responsible for preventing Ca^++ ^influx.

Calreticulin (Cal) is a protein that binds to Ca^++ ^and prevents apoptosis due to Ca^++ ^overload. In the E-D model, the position was taken that Cal was upregulated by mAR, however, in the extended E-D model, the position is that Cal is upregulated by iAR. In the fully grown prostate, F slightly inhibited Cal production [37], which is consistent with iAR upregulating Cal. It is not clear what the affinity of T-BSA, T, or DHT is to mAR, but equal concentrations of these hormones resulted in identical levels of apoptosis in the PC cell line DU145 after 24 hours [26]. This is consistent with T and DHT binding to mAR with somewhat similar affinities, but further research is needed. Since DHT binds with greater affinity than T to iAR, then the decrease in Cal production in the presence of F is consistent with iAR upregulating Cal. Further research is needed to determine what effect mAR has on Cal regulation.

### Prevention

In designing protocols for preventing BC and PC, every effort should be made to avoid potential long term side effects, while still increasing R_D _as much as possible, so that R_G _< R_D _for any early stage cancer cells that may already be present. This means that, for safety concerns, no drugs should be used which block hormone receptors, since, until proven otherwise, it must be assumed that every hormone receptor has some purpose in the overall health of the body. Also, hormone levels should be kept within their physiological limits until evidence is produced that shows that it is safe to go outside of those limits. Within these constraints, the goal is to maximize the production of apoptotic proteins upregulated by mAR and to minimize the production of bcl-2.

One way to minimize bcl-2 production would be to maximize the activity of PRB and mPR while minimizing the activity of PRA. However, since no hormone has yet been discovered that does this, then P has to be considered instead. P should be increased to the maximum safe physiological amount appropriate for the gender of the individual being treated, unless testing shows a genetic makeup that results in an increase in bcl-2 in the breast or prostate epithelial cells in response to P, such as in the case of BRCA1 or BRCA2 mutations.

Another way to minimize Bcl-2 would be by using a hormone that binds preferentially to ER-β over ER-α and mER. Estriol (E3) has an affinity for ER-β which is 3.5 times greater than for ER-α, E2 has an equal affinity for ER-α and ER-β, and estrone has an affinity for ER-α which is 5 times greater than for ER-β[38]. This is consistent with E3 being the preferred ligand for ER-β. However, E3 binds to ER-β only 35% as strongly as E2 does. More research is needed to determine whether E3, possibly in combination with a drug to block Aro in order to minimize the local level of E2, would be helpful or not.

In order to maximize the production of apoptotic proteins upregulated by mAR, binding to mAR should be increased as much as possible and binding to iAR should be decreased as much as possible. Since T and DHT seem to have similar affinities to mAR, whereas DHT has an affinity to iAR which is five times greater than T, then high T and low DHT (HTLD) should create the desired imbalance. Therefore, the serum level of bioavailable T should be increased to the maximum safe physiological level appropriate for the gender of the individual being treated, while the serum level of DHT should be decreased to the minimum physiological level necessary for maintaining good health. Since T can be converted to E2 by Aro, the level of E2 should be monitored and kept within normal or low normal physiological levels. In addition to increasing the apoptotic proteins upregulated by mAR, this protocol should increase Ca^++ ^influx and decrease Cal production, all of which should increase R_D_.

When LNCaP tumors were transplanted into nude mice, four weeks of T-BSA administration resulted in a 60% reduction in tumor volume when compared to BSA administration alone [26]. Also, when LNCaP tumors were transplanted into nude mice, treatment with T plus F following intermittent androgen ablation resulted in no change or a decrease in tumor volume for 41% of the mice, as compared to 10% of the mice treated with T alone [39]. This is consistent with the greatest increase in R_D _occurring after full agonism of mAR along with no agonism of iAR. However, if there is a great amount of agonism of mAR along with a small amount of agonism of iAR, then there would still be an increase in R_D _for PC if the imbalance in the binding to the androgen receptors is great enough. Using F to prevent DHT creates such an imbalance, since T has an affinity which is five times less than that of DHT to iAR. This raises the possibility that HTLD would result in R_G _< R_D _for most early stage BC or PC cells.

The active metabolite of vitamin D is 1,25(OH)_2_D_3 _(calcitriol). When calcitriol bound to the vitamin D receptor (VDR), it inhibited growth and upregulated AS3 in a number of PC cell lines [40] and increased cell death in BC [41] and PC [42]. Calcitriol caused cell death primarily by a caspase-independent mitochondrial pathway in BC [41] and in PC [42]. Also, bcl-2 inhibited the cell death caused by calcitriol in BC [41] and in PC [42]. In some PC cell lines, bicalutamide repressed the inhibition in growth and upregulation of AS3 cause by calcitriol [40]. This is consistent with the upregulation of AS3 by calcitriol being dependent on properly functioning iAR. As part of the prevention protocol, the serum level of calcitriol should be increased to the maximum safe physiological level. This may decrease R_G _in BC and PC, and if the level of bcl-2 is low enough, may increase R_D_.

HTLD will have different effects with regards to bcl-2 production for BC and PC. For BC, the increased amount of T binding to mAR will result in a decrease in bcl-2 due to increased downregulation. However, the decreased amount of DHT binding to iAR will result in less downregulation of bcl-2 production and therefore an increase in bcl-2. Therefore, there should not be a dramatic increase in bcl-2 for BC as a result of HTLD.

For PC, however, the increased amount of T binding to mAR will result in an increase in bcl-2 due to increased upregulation and the decreased amount of DHT binding to iAR will also result in an increase in bcl-2 due to decreased downregulation. Therefore, for preventing PC, more care must be used to decrease bcl-2 in other ways, if possible. Also, large quantities of foods which contain components which bind to ER-β with less than full agonism should be avoided. This is because such components might interfere with E2 binding to ER-β and thus reduce the downregulation of bcl-2. For example, genistein, the main isoflavone found in soy, increased bcl-2 in the BC cell line MCF-7 [43].

Anecdotally, some men with PC who were taking 5AR2 inhibitors following ADT exhibited consistent increases in PSA values associated with the introduction of large doses of genistein, soy, tofu, modified citrus pectin, or flaxseed into a pre-existing diet. Often this change in PSA trajectory could be reversed by stopping that nutritional product [44]. This is consistent with the use of 5AR2 inhibitors resulting in an increase in bcl-2 as well as a decrease in the downregulation of apoptotic proteins upregulated by mAR. Ordinarily, the decrease in the downregulation of apoptotic proteins has more of an effect than the increase in bcl-2, as evidenced by the apoptotic effect of T-BSA. However, if large amounts of food are ingested which bind preferentially to ER-β, then the overall increase in bcl-2 may decrease R_D _more than the apoptotic proteins increase R_D_. This would be expressed by a more rapid population growth, which would account for the observed increase in PSA for those men taking 5AR2 inhibitors. Pharmacological amounts of genistein induced apoptosis in PC cell lines by a process independent of its binding to estrogen receptors [45]. Therefore, it is likely that physiological amounts of genistein increase R_D _to some extent. However, when 5AR2 inhibitors are used in conjunction with genistein, the overall increase in bcl-2 that results may more than offset the anticancer effects of genistein, if any PC cells are already present. If no PC cells are present, then ingesting phytoestrogens should help prevent PC, since the phytoestrogens should interfere to some degree with the ability of E2 to upregulate telomerase. Pharmacological levels of genistein did suppress telomerase activity in the PC cell lines LNCaP and DU-145 [46].

Although in using the HTLD protocol for preventing PC, the hormones would be kept within physiological levels, there is still the possibility that long term use of this protocol may have some health consequences unrelated to PC. Lean elderly men and women who have Alzheimer's disease (AD) had lower bioavailable levels of T than those without AD [47]. This might be due to AD causing a drop in the level of bioavailable T, or by the low bioavailable level of T increasing the likelihood of developing AD. T downregulated β-amyloid peptides *in vitro *[48], and β-amyloid is considered to be crucial in the pathogenesis of AD [49]. This increases the likelihood that the decreased levels of bioavailable T were responsible for the increased incidences of AD. If so, then the HTLD protocol for preventing BC and PC may also be helpful in preventing AD.

In a five year study for male veterans over 40 years of age, those with low levels of T had a mortality rate of 34.9% as compared to 20.1% for those with normal levels of T [50]. Low levels of T were defined as a level of total T below 250 ng/dL or a level of free T below 0.75 ng/dL. This raises the possibility that the HTLD protocol for preventing PC might also result in increased longevity for men. It is not yet known what the relationship between T and longevity is for women. More research is needed to fully identify all beneficial and detrimental effects that may result from using the HTLD protocol in men and in women.

In summary, the protocol for preventing both BC and PC involves obtaining gender appropriate maximum safe physiological levels of bioavailable T, maximum safe physiological level of calcitriol, minimum safe physiological level of DHT and normal level of E2. Maximum safe physiological levels of P should be added except for those individuals whose genetic makeup would not benefit from P. If further research should determine that E3 is helpful, then maximum safe physiological levels of E3 should be added. Also, ingesting large quantities of foods which are known to bind to ER-β with less than full agonism should be avoided. Other factors, such as nutritional supplements or lifestyle changes which are shown to reduce the incidence of BC and PC, can also be included. Table 3 shows the effects of the HTLD protocol.

**Table 3:** HTLD Protocol

**Treatment**	**Results**	**Effects**
High T	↑apoptotic proteins ↓bcl-2 (BC only) ↑bcl-2 (PC only) ↓AS3 ↑Ca^++ ^influx	↑ R_D_ ↑ R_D_ ↓ R_D_ ↑ R_G_ ↑ R_D_
Low DHT	↑apoptotic proteins ↑bcl-2 ↓AS3 ↑Ca^++ ^influx ↓calreticulin	↑ R_D_ ↓ R_D_ ↑ R_G_ ↑ R_D_ ↑ R_D_
High P	↓bcl-2 (favorable genetics)	↑ R_D_
High calcitriol	↑AS3 ↑kill mitochondria	↓ R_G_ ↑ R_D_
Lowering phytoestrogens	↓bcl-2	↑ R_D_

It is possible that the HTLD protocol might be ineffective or even harmful depending on the mutations that may be in some of the BC or PC already present. For example, if there is a mutation in PC that prevents mAR from upregulating apoptotic proteins but still allows it to upregulate bcl-2, then the HTLD protocol would be harmful. The earlier this protocol is started, the less likely that any such adverse mutations would be present.

An alternative strategy for prevention would involve all of the steps listed above, but in place of maximizing the upregulation of apoptotic proteins by mAR through HTLD, instead minimize the amount of bcl-2 present and rely on the high serum level of calcitriol to maximize apoptosis. For BC, the gender appropriate maximum physiological level of bioavailable T and DHT or high T and high D (HTHD) would reduce the production of bcl-2 in comparison to HTLD, since DHT downregulates bcl-2. This decrease in bcl-2 should increase the likelihood that calcitriol would increase R_D_. However, it would also eliminate the imbalance that should upregulate the apoptotic proteins associated with mAR, which should result in a decrease in R_D_. Further research is needed to determine whether HTLD or HTHD is more effective in preventing BC. For HTHD, there is no need to avoid ingesting phytoestrogens, since no 5AR2 inhibitors would be present and therefore no decrease in bcl-2. Table 4 shows the effects of the HTHD protocol.

**Table 4:** HTHD Protocol

**Treatment**	**Results**	**Effects**
High T	↑apoptotic proteins ↓bcl-2 (BC only) ↑bcl-2 (PC only) ↓AS3 ↑Ca^++ ^influx	↑ R_D_ ↑ R_D_ ↓ R_D_ ↑ R_G_ ↑ R_D_
High DHT	↓apoptotic proteins ↓bcl-2 ↑AS3 ↓Ca^++ ^influx ↑calreticulin	↓ R_D_ ↑ R_D_ ↓ R_G_ ↓ R_D_ ↓ R_D_
High P	↓bcl-2 (favorable genetics)	↑ R_D_
High calcitriol	↑AS3 ↑kill mitochondria	↓ R_G_ ↑ R_D_

For PC, the minimum safe physiological level of bioavailable T and the maximum safe physiological level of DHT or low T and high D (LTHD) should reduce bcl-2 even more than HTHD does, assuming that maximum agonism of mAR is not achieved with the maximum safe physiological level of DHT alone. This is because reducing the level of T would reduce the overall amount of androgen available to bind to mAR and mAR upregulates bcl-2 in PC. Further research is needed to determine whether HTLD or LTHD is more effective in preventing PC. Also, for LTHD there is no need to avoid ingesting phytoestrogens. Table 5 shows the effects of the LTHD protocol.

**Table 5:** LTHD Protocol

**Treatment**	**Results**	**Effects**
Low T	↓apoptotic proteins ↑bcl-2 (BC only) ↓bcl-2 (PC only) ↑AS3 ↓Ca^++ ^influx	↓ R_D_ ↓ R_D_ ↑ R_D_ ↓ R_G_ ↓ R_D_
High DHT	↓apoptotic proteins ↓bcl-2 ↑AS3 ↓Ca^++ ^influx ↑calreticulin	↓ R_D_ ↑ R_D_ ↓ R_G_ ↓ R_D_ ↓ R_D_
High P	↓bcl-2 (favorable genetics)	↑ R_D_
High calcitriol	↑AS3 ↑kill mitochondria	↓ R_G_ ↑ R_D_

### Treatment

When treating BC or PC systemically, the goal should be to minimize bcl-2 and to maximize apoptotic proteins, without regards to long term health risks. If the genetic makeup of the BC or PC were known, then treatments could be individually designed for optimal effectiveness. However, due to the heterogeneous nature of BC and PC, care must be taken to consider all possible mutations and, whenever possible, to avoid using any treatment that would ever decrease R_D_. Ideally, if the initial treatment is successful, then treatment can eventually be changed to one of the preventative protocols described previously.

Systemic hormonal manipulation is currently being used, to a limited extent, for both PC and BC. In PC, the form of systemic hormonal manipulation currently being used is ADT. During ADT, downregulation of Cal coupled with Ca^++ ^influx may lead to apoptosis [36]. For prostate cells, the level of apoptosis in the absence of androgen is the same as that in the presence of androgen if ionophores are used to cause sufficiently high Ca^++ ^influx [51]. In the absence of androgen, the increased amount of apoptosis could be reduced by up to 70% through the use of Ca^++ ^channel blockers. This is all consistent with Ca^++ ^overload being the cause of apoptosis during ADT. When ADT is administered, typically nothing is done to maximize the upregulation of apoptotic proteins or to maximize the downregulation of bcl-2.

For BC which has ER-α present, currently systemic hormonal manipulation is aimed at reducing the binding of E2 to ER-α. This is accomplished either by using tamoxifen, in order to block the binding to ER-α, or anastrozole, which is an antagonist to Aro, in order to reduce the amount of E2 present in the BC cells. In both cases, bcl-2 production should be reduced, since ER-α upregulates bcl-2. However, nothing is done to utilize any of the other hormone receptors to further reduce bcl-2 production and nothing is done to maximize the production of apoptotic proteins.

There are a number of options available in searching for the optimum treatment protocol. One consideration is whether or not localized treatment, such as surgery, should be done initially for BC or PC. It is known that if surgery does not remove all cancer cells, the remaining cancer cell population doubles at a quicker rate than it did before the surgery [52]. Increased angiogenesis is one of the proposed explanations for this. If this increased rate of population growth is shown to be at all due to an increase in R_G_, then it would be more difficult to use systemic treatment to achieve R_G _< R_D _following surgery. If systemic hormonal treatment can be shown to be sufficiently effective in early stage treatment, it is possible that localized treatment may not be necessary. However, systemic hormonal treatment must be continued indefinitely in case any BC or PC cells remain, whereas surgery has the possibility of being curative. There is also the possibility that surgery might remove cancer cells that have already mutated to the point that systemic treatment would be ineffective on them, so that surgery followed by systemic treatment might be successful whereas systemic treatment without surgery might be a failure. More research is needed to clarify this point.

Men with stage T1–T2 PC, with a mean prostate specific antigen (PSA) of 13.5, whose initial treatment was radical prostatectomy (RP) had a PC specific death rate of 4.6% and a 10.1% rate of distant metastases after a median of 6.2 years [53]. Men with stage T1–T3 PC, with a mean PSA of 11.1, whose initial treatment was 13 months of ADT utilizing a luteinizing hormone-releasing hormone agonist to reduce T production, plus an antiandrogen to block iAR, plus F to reduce DHT by inhibiting 5AR2, followed after those 13 months by continual F only, had a PC specific death rate of 0.6% [54] and a 0.6% rate of distant metastases [44] after a median of 6.2 years. All of the men in this study were told to avoid ingesting large amounts of phytoestrogens [44]. This raises the possibility that initial systemic treatment may be a viable alternative to local treatments for PC.

While this systemic treatment compares quite favourably with RP, it is possible to make improvements during ADT based on the extended E-D model. Maximum antagonism of mAR and iAR should be used. In order to obtain the lowest level of bcl-2 from the non-androgen receptors (LBNAR), maximum antagonism of ER-α, mER, and PRA should be used, as well as maximum agonism of ER-β, PRB, and mPR. P should be used only in the presence of a drug that blocks the conversion of P to T, since P is able to be converted to T [55]. Also, maximum agonism of VDR (MAV) should be used in order to increase R_D _by killing mitochondria.

Incorporating these modifications should minimize the amount of bcl-2 present while maintaining the apoptotic forces of ADT. Further research is needed to verify that the LBNAR protocol maintains Ca^++ ^influx coupled with the absence of Cal which is known to occur in ADT without LBNAR. It is possible that some of the non-androgen receptors are involved in the regulation of Ca^++ ^influx and Cal. For example, there is evidence [56] that mER upregulates Ca^++ ^influx in the PC cell line LNCaP. Table 6 shows the effects of the enhanced ADT treatment.

**Table 6:** Enhanced ADT Treatment

**Treatment**	**Results**	**Effects**
Maximum antagonism of mAR	↓apoptotic proteins ↑bcl-2 (BC only) ↓bcl-2 (PC only) ↑AS3 ↓Ca^++ ^influx	↓ R_D_ ↓ R_D_ ↑ R_D_ ↓ R_G_ ↓ R_D_
Maximum antagonism of iAR	↑apoptotic proteins ↑bcl-2 ↓AS3 ↑Ca^++ ^influx ↓calreticulin	↑ R_D_ ↓ R_D_ ↑ R_G_ ↑ R_D_ ↑ R_D_
Maximum antagonism of ER-α	↓bcl-2	↑ R_D_
Maximum agonism of ER-β	↓bcl-2	↑ R_D_
Maximum antagonism of mER	↓bcl-2	↑ R_D_
Maximum antagonism of PRA	↓bcl-2	↑ R_D_
Maximum agonism of PRB	↓bcl-2	↑ R_D_
Maximum agonism of mPR	↓bcl-2	↑ R_D_
Maximum calcitriol	↑kill mitochondria	↑ R_D_

Following ADT, there should be maximum agonism of mAR coupled with maximum antagonism of iAR, or all mAR no iAR (AMNI). AMNI should maximize the production of the apoptotic proteins upregulated by mAR, and should increase the level of bcl-2, since mAR upregulates bcl-2 and iAR downregulates bcl-2. There should also be increased Ca^++ ^influx and decreased production of Cal. It is possible that AMNI will not result in the same level of apoptosis from Ca^++ ^overload as what is seen in ADT, since other receptors besides iAR and mAR may be involved in Ca^++ ^influx and Cal production. LBNAR should be added to minimize bcl-2 production. MAV should also be added to AMNI. This should increase R_D _if the overall level of bcl-2 is low enough, but should not increase AS3 due to the antagonism of iAR. The optimum length of time to maintain this treatment needs to be determined. Table 7 shows the effects of the AMNI treatment.

**Table 7:** AMNI Treatment

**Treatment**	**Results**	**Effects**
Maximum agonism of mAR	↑apoptotic proteins ↓bcl-2 (BC only) ↑bcl-2 (PC only) ↓AS3 ↑Ca^++ ^influx	↑ R_D_ ↑ R_D_ ↓ R_D_ ↑ R_G_ ↑ R_D_
Maximum antagonism of iAR	↑apoptotic proteins ↑bcl-2 ↓AS3 ↑Ca^++ ^influx ↓calreticulin	↑ R_D_ ↓ R_D_ ↑ R_G_ ↑ R_D_ ↑ R_D_
Maximum antagonism of ER-α	↓bcl-2	↑ R_D_
Maximum agonism of ER-β	↓bcl-2	↑ R_D_
Maximum antagonism of mER	↓bcl-2	↑ R_D_
Maximum antagonism of PRA	↓bcl-2	↑ R_D_
Maximum agonism of PRB	↓bcl-2	↑ R_D_
Maximum agonism of mPR	↓bcl-2	↑ R_D_
Maximum calcitriol	↑kill mitochondria	↑ R_D_

Since the AMNI treatment may fail against PC with mutated mAR that is unable to upregulate apoptotic proteins, it should be followed by a treatment of maximum antagonism of mAR along with maximum agonism of iAR, or no mAR all iAR (NMAI). NMAI should increase the production of AS3 upregulated by iAR to stop cell proliferation, and should lower bcl-2 levels. LBNAR and MAV should also be added to NMAI. In this case, MAV should decrease R_G _by increasing the production of AS3 and increase R_D_, since, as opposed to AMNI, NMAI should reduce bcl-2 production in PC. Table 8 shows the effects of the NMAI treatment. Mutations in the iAR that bind to E2 and P, such as exists in LNCaP, would protect the cells against AMNI, but should be vulnerable to NMAI. NMAI should be much less effective against PC with non-functioning iAR, but such cells should have already undergone apoptosis from the AMNI treatment. Incorporating both AMNI and NMAI should maximize overall PC cell death.

**Table 8:** NMAI Treatment

**Treatment**	**Results**	**Effects**
Maximum antagonism of mAR	↓apoptotic proteins ↑bcl-2 (BC only) ↓bcl-2 (PC only) ↑AS3 ↓Ca^++ ^influx	↓ R_D_ ↓ R_D_ ↑ R_D_ ↓ R_G_ ↓ R_D_
Maximum agonism of iAR	↓apoptotic proteins ↓bcl-2 ↑AS3 ↓Ca^++ ^influx ↑calreticulin	↓ R_D_ ↑ R_D_ ↓ R_G_ ↓ R_D_ ↓ R_D_
Maximum antagonism of ER-α	↓bcl-2	↑ R_D_
Maximum agonism of ER-β	↓bcl-2	↑ R_D_
Maximum antagonism of mER	↓bcl-2	↑ R_D_
Maximum antagonism of PRA	↓bcl-2	↑ R_D_
Maximum agonism of PRB	↓bcl-2	↑ R_D_
Maximum agonism of mPR	↓bcl-2	↑ R_D_
Maximum calcitriol	↑AS3 ↑kill mitochondria	↓ R_G_ ↑ R_D_

For BC, the initial treatment should also be maximum antagonism of mAR and iAR along with LBNAR and MAV. This should be effective assuming that iAR upregulates Cal and downregulates Ca^++ ^influx as it does for PC. Next, the AMNI protocol along with LBNAR and MAV should be done. This would have similar benefits as was described for PC, although because mAR downregulates bcl-2 in BC, as opposed to upregulating it in PC, the R_D _would be expected to be greater, since the level of bcl-2 should be lower. Just as in PC, the NMAI protocol along with LBNAR and MAV should be done next. This should have an equivalent effectiveness against BC as it had against PC. An additional protocol to consider for BC would be to use maximum agonism of mAR and of iAR, or all mAR all iAR (AMAI). When LBNAR and MAV are added, this should have a bcl-2 level lower than for any of the other protocols, but it would then be dependent on calcitriol killing mitochondria to increase R_D _and upregulating AS3 to decrease R_G_. Table 9 shows the effects of the AMAI treatment.

**Table 9:** AMAI Treatment

**Treatment**	**Results**	**Effects**
Maximum agonism of mAR	↑apoptotic proteins ↓bcl-2 (BC only) ↑bcl-2 (PC only) ↓AS3 ↑Ca^++ ^influx	↑ R_D_ ↑ R_D_ ↓ R_D_ ↑ R_G_ ↑ R_D_
Maximum agonism of iAR	↓apoptotic proteins ↓bcl-2 ↑AS3 ↓Ca^++ ^influx ↑calreticulin	↓ R_D_ ↑ R_D_ ↓ R_G_ ↓ R_D_ ↓ R_D_
Maximum antagonism of ER-α	↓bcl-2	↑ R_D_
Maximum agonism of ER-β	↓bcl-2	↑ R_D_
Maximum antagonism of mER	↓bcl-2	↑ R_D_
Maximum antagonism of PRA	↓bcl-2	↑ R_D_
Maximum agonism of PRB	↓bcl-2	↑ R_D_
Maximum agonism of mPR	↓bcl-2	↑ R_D_
Maximum calcitriol	↑AS3 ↑kill mitochondria	↓ R_G_ ↑ R_D_

More research is needed to determine the effectiveness of these treatments and the optimal time to maintain each treatment. For both PC and BC, if the treatments are successful then one of the preventative protocols can then be used.

## Discussion

The protocols given for preventing and treating BC and PC are merely suggestions based on the properties of the extended E-D model. There are other possible alternatives that can be tried. In the case of prevention, it is possible that raising T to higher than physiological levels when using HTLD may have beneficial effects. Individuals with mutations in BRCA1 or BRCA2 may want to start a preventative protocol at an earlier age. A protocol for prevention may also be applied to patients after they initially receive localized treatment, such as surgery or radiation. Changes in lifestyle that are shown to be useful against BC and PC, such as diet and exercise, can be added to the protocols for prevention and treatment.

BC and PC are complex diseases, and the properties of hormone receptors described in the extended E-D model represent a foundation which can be built on to better understand both diseases. Bcl-2 is chosen as the main antiapoptotic protein to focus on in this model because it has been shown to be extremely powerful. It prevented apoptosis caused by calcitriol in BC [41] and in PC [42]. Also, bcl-2 is known to be able to prevent apoptosis caused by Fas [57] and by Bad [20]. Just by increasing bcl-2, using a vector of cDNA, LNCaP turned into an androgen independent PC cell line [58]. This is consistent with bcl-2 protecting against the apoptosis caused by ADT. The increased chance of developing BC and PC in individuals with either the BRCA1 or BRCA2 mutation is consistent with the increased bcl-2 caused by the elimination of PRB being partly responsible for the increased incidence of cancer. Also, assuming that there is a purpose in the pattern of which hormone receptors upregulate bcl-2 and which downregulate bcl-2, then it is possible that the same pattern may apply to other anti-apoptotic proteins as well.

If iAR is not functional, then apoptotic proteins will be upregulated by mAR in both BC and PC. In BC, bcl-2 will also be downregulated, helping to further increase R_D_, whereas, in PC, bcl-2 will be upregulated. In PC, this creates a situation in which the same hormone receptor exhibits one property that increases the chance of apoptosis and another that decreases the chance of apoptosis. Ordinarily, apoptosis will occur if a sufficient quantity of androgen is present, as evidenced by the fact that T-BSA resulted in a 60% reduction in tumor size of LNCaP transplanted into nude mice after one month [26]. Since mAR downregulates bcl-2 in BC, less T should be needed in order to achieve apoptosis in BC than in PC. This means that for men, BC should be much less likely to occur than in women, in part because of the higher levels of T that men possess when compared to women. In fact, men rarely develop BC. However, the incidence of BC increases [59] in men who suffer from disorders related to hypoandrogenism.

Although telomerase activity may immortalize cells, it is not sufficient by itself to produce cancer as evidenced by the fact that the tissue cultures with telomerase activity did not become cancerous [2]. It is believed that there are six properties that a cell must acquire in order to become cancerous [5]. These properties are self-sufficiency in growth signals, insensitivity to antigrowth signals, evading apoptosis, limitless replicative potential, sustained angiogenesis, and tissue invasion and metastasis. Such changes would confer a great selective advantage when they occur in immortalized cells growing within an organ confined space. However, it is not clear that such changes would confer much of an advantage to immortalized cells growing in a tissue culture.

A key prediction of the extended E-D model is that HTLD will increase R_D _in both BC and PC. One experiment [39] that highlights the power of this treatment used LNCaP cells transplanted into nude castrated mice, then treated with T plus F following intermittent androgen ablation. The change in tumor volume ended up being around 5 times less than that when continual androgen ablation was used. The proteins that are rapidly produced, presumably upregulated by mAR, are only observed to be present for a few hours following the addition of T plus F to end the androgen ablation. In the absence of androgen ablation, production of these proteins following the addition of T plus F is not observed. This raises the possibility that not just DHT, but also T binding to iAR is sufficient to completely downregulate the proteins upregulated by mAR, so that no net production of these proteins occurs. Another possibility is that some small amount of apoptotic proteins upregulated by mAR is continually being produced in the presence of HTLD, and the accumulation of these proteins might be responsible for apoptosis. Also, due to the low DHT, there might be increased Ca^++ ^influx along with lowered production of Cal, which might increase R_D _as well.

If the observed apoptosis was totally due to the apoptotic proteins upregulated by mAR when initially unopposed by downregulation from iAR, then the amount of apoptosis should be about the same for T and DHT. However, when T alone was used to end androgen ablation, the result was an average increase of 128% in tumor volume. This was much worse than the average increase of 23% in tumor volume observed when T plus F was used to end androgen ablation. Also, considering that LNCaP is an ADPC cell line, the addition of T should have resulted in an increase in tumor volume much greater than that observed with continual androgen ablation. The fact that continual androgen ablation had an average increase of 114% in tumor volume means that using T alone after androgen ablation was only a little worse than continual androgen ablation. This difference suggests that the initial increase in apoptotic proteins that occurred when the faster acting mAR was active, but the slower acting iAR was not yet active, might be responsible for the better than expected results for T alone.

Another possibility is that the effectiveness of HTLD was due to the low DHT caused by F, either because of a decrease in R_G _or an increase in R_D_. However, when androgen ablation plus F was used, the average increase in tumor volume was 91%, which was a bit better than continual androgen ablation, but still much worse than HTLD. As a result, the benefits observed from using T plus F are consistent with an initial surge followed by a slow continual release of apoptotic proteins due to the imbalance in the binding of T to both mAR and iAR as compared to the binding of T to mAR and DHT to iAR.

In order to determine the best protocol for preventing BC and PC, the HTLD, HTHD, and LTHD protocols should be examined for their efficacy in increasing R_D _for BC and PC in animal studies as well as with various cell lines. Since it is assumed that some BC or PC cells may be present before treatment is started, it is important that the protocol used have the ability to cause apoptosis or inhibit the growth of existing BC or PC. Also, in addition to a protocol's effectiveness in preventing BC and PC, its impact on quality of life must be considered. Since the protocol will be used for long term, a significant improvement in quality of life might offset a slightly inferior effectiveness in preventing BC and PC. It is also possible that alternating between the preventative protocols might be more effective than maintaining just one. More research is needed to examine these possiblities.

In considering the best protocol for treating BC and PC, the theoretical ideals were given, with no consideration to the side effects of the treatment or whether the necessary drugs existed and were available for human use. In practice, both of these must be considered and modifications must be made, while trying to stay as close to the theoretical ideal as possible. For example, it is known that high levels of calcitriol cause hypercalcemia, but research is being done [60] to develop vitamin D analogues which are capable of being agonists to VDR while not producing hypercalcemia.

For a model to be an accurate reflection of reality, it should be able to explain all observed experimental results. The extended E-D model can explain some experimental findings in a straightforward manner. One example would be the fact that both exogenous T and E2 must be given to Noble rats in order to reproducibly induce PC [61]. The high level of E2 would increase telomerase activity directly in the prostate epithelial cells, without the need for Aro activity. If T were not also added, then the E2 would suppress the production of T [62], resulting in the high R_D _typical of ADT. If T were used without E2, then telomerase activity would occur when Aro was activated, which would produce high local levels of E2 and telomerase activity. However, for prostate epithelial cells to express Aro activity requires either a mutation or presumably a failure to methylate the portion of DNA containing the Aro gene. Therefore, the fact that the percentage of Noble rats that develop PC when exposed just to exogenous T is much lower than the percentage that develop PC when exposed to exogenous T plus E2 is consistent with the extended E-D model.

Another example is that mammary epithelial proliferation in ovariectomized rhesus monkeys occurred after three days of treatment with tamoxifen [63]. This is consistent with OHT upregulating telomerase activity in breast epithelial cells by acting as an agonist to ER-α homodimers, just as it does for PC. The long term effect of tamoxifen use would depend on the values of R_G _and R_D _that result.

One example that is more problematic for the extended E-D model to explain is the relationship between serum levels of T and incidence of BC and PC. The extended E-D model would predict that there is a separate threshold level of bioavailable T for BC and PC, above which R_G _< R_D_. For levels below this threshold, R_G _> R_D _and if BC or PC develops, then it can proliferate. As bioavailable levels of T decrease further, R_D _should also decrease. This should not increase the incidence of BC or PC, but should increase the aggressiveness of the disease. For BC, this is because lower agonism of mAR would result in less apoptotic proteins being upregulated, and lower agonism of iAR would result in less AS3 being upregulated. In addition, there should be higher levels of bcl-2, since both mAR and iAR downregulate bcl-2. The same factors would explain the increase in PC, except that in prostate epithelial cells mAR upregulates bcl-2 whereas iAR downregulates bcl-2. This would result in less of an increase in bcl-2 than occurs in breast epithelial cells. Lower levels of T were associated with worsening clinical staging, worsening histological staging, and more poorly differentiated adenocarcinomas for PC [64].

However, higher levels of free T are correlated with slightly increased incidences of BC [65] and PC [66]. The higher level of free T would mean that the intracellular level of T should be higher than for those individuals with lower levels of free T. The higher intracellular level of T should result in higher local levels of E2 if Aro activity is present. The higher level of E2 might lead to higher telomerase activity and possibly an increase in R_G_, making it more likely that R_G _> R_D _for some individuals. Another possibility is that the higher level of free T results in higher local levels of DHT. This increase in DHT should result in increased agonism of iAR which should result in more downregulation of the apoptotic proteins upregulated by mAR. If ordinarily apoptosis is caused in part by the slow accumulation of these apoptotic proteins, then the increased downregulation by iAR should result in a decrease in R_D_, increasing the possibility that R_G _> R_D_. Also, if the level of free T becomes low enough, then even in the presence of Aro activity, the local level of E2 that results would be too low to upregulate telomerase activity, removing Aro activity as a cause for BC or PC.

More research is needed to test the properties of the extended E-D model. Experiments concentrating on individual hormone receptors are essential. The extended E-D model can be expanded to include how hormone receptors upregulate or downregulate other proapoptotic and antiapoptotic proteins as they are discovered.

## Conclusion

BC and PC appear to be functionally identical, but there are slight differences in the way each disease achieves that functionality. The most striking difference between the two diseases is the difference in the properties of their mAR. In both BC and in PC, apoptosis occurs following the loss of functionality of their iAR. However, since women have much lower levels of T than men do, in order to maintain the identical functionality it is necessary for mAR to be more effective in inducing apoptosis in BC than in PC, which in fact appears to be the case. For both BC and PC, mAR upregulates apoptotic proteins, but for BC, mAR also downregulates bcl-2, whereas for PC, mAR upregulates bcl-2.

BC and PC are complex diseases, but by focusing on the properties of the individual hormone receptors, it is possible to develop systemic protocols for prevention and treatment. Such protocols can be augmented by any lifestyle changes, such as diet and exercise, which may be shown to be helpful.

## Competing interests

The author(s) declare that they have no competing interests.
